# Alteration of Maternal Serum Ferritin in Pregnancy and Maternal-fetal Infections: A retrospective cohort study

**DOI:** 10.12669/pjms.40.7.9160

**Published:** 2024-08

**Authors:** Xing Liao, Xiaoyan Xiu, Guizhen Xu, Ling Wu, Zhuanji Fang, Huihui Huang

**Affiliations:** 1 Xing Liao, Department of Pediatrics, Fujian Children’s Hospital, Fuzhou, Fujian Province 350001, China. Fujian Maternity and Child Health Hospital; College of Clinical Medicine for Obstetrics and Gynecology and Pediatrics,Fujian Medical University, Fuzhou, Fujian Province 350001, China; 2 Xiaoyan Xiu Department of Obstetrics, Fujian Maternity and Child Health Hospital; College of Clinical Medicine for Obstetrics and Gynecology and Pediatrics, Fujian Medical University, Fuzhou, Fujian Province 350001, China; 3 Zhuanji Fang Department of Obstetrics, Fujian Maternity and Child Health Hospital; College of Clinical Medicine for Obstetrics and Gynecology and Pediatrics, Fujian Medical University, Fuzhou, Fujian Province 350001, China; 4 Guizhen Xu Department of Obstetrics, Fujian Maternity and Child Health Hospital; College of Clinical Medicine for Obstetrics and Gynecology and Pediatrics, Fujian Medical University, Fuzhou, Fujian Province 350001, China; 5 Ling Wu, Department of Pediatrics, Fujian Children’s Hospital, Fuzhou, Fujian Province 350001, China. Fujian Maternity and Child Health Hospital; College of Clinical Medicine for Obstetrics and Gynecology and Pediatrics, Fujian Medical University, Fuzhou, Fujian Province 350001, China; 6 Huihui Huang Department of Obstetrics, Fujian Maternity and Child Health Hospital; College of Clinical Medicine for Obstetrics and Gynecology and Pediatrics, Fujian Medical University, Fuzhou, Fujian Province 350001, China

**Keywords:** Neonatal Sepsis, Serum Ferritin, Chorioamnionitis, Maternal-fetal Infections, Placental Pathology

## Abstract

**Objectives:**

To investigate the association of altered serum ferritin during pregnancy with chorioamnionitis and neonatal sepsis.

**Methods:**

This retrospective cohort study included 78,521 pregnant women who attended antenatal check-ups at maternal and child health centers in Fujian Province, China. Study lasted from January 2014 to January 2019. A total of 59,812 pregnant women were followed up. Patients with suspected infection before the delivery were selected and divided into the chorioamnionitis and non-chorioamnionitis groups according to placental pathology. Differences in late and early pregnancy serum ferritin between the two groups were compared. Multiple logistics regression was used to adjust for confounding factors and to analyze the association between serum ferritin changes and pregnancy outcomes. Importance of altered serum ferritin during pregnancy was assessed by receiver operating characteristic (ROC) curve and net reclassification index (NRI).

**Results:**

Clinical records of 8506 pregnant women were included in the study. there were 1010 (11.9%) cases of confirmed chorioamnionitis and 263 (3.1%) cases of neonatal sepsis. There was a significant difference in maternal serum ferritin changes between the groups with and without chorioamnionitis. No significant difference was detected in cases with or without neonatal sepsis. Multiple logistic regressions, corrected for confounding factors yielded similar conclusions. Maternal serum ferritin difference NRI 12.18% (p = 0.00014) was similar to the ROC results in predicting the occurrence of chorioamnionitis.

**Conclusion:**

Differential serum ferritin during pregnancy may predict chorioamnionitis but does not correlate well with neonatal sepsis.

## INTRODUCTION

Iron is essential for various physiological processes in the human body, including immune function. However, excess iron can be harmful and has been associated with increased susceptibility to infections.^1^ Levels of serum ferritin, a protein that stores iron, are often used as a biomarker of iron status in clinical settings.^2^-^4^ Elevated serum ferritin levels have been associated with a variety of diseases, including iron overload syndrome, chronic liver disease, and malignancies. Moreover, recent studies suggests that serum ferritin levels are significantly elevated during viral and bacterial infectious diseases.^5^ Elevated serum ferritin levels have been found to be associated with severe clinical outcomes in infectious diseases such as COVID-19, sepsis, and viral hepatitis. Therefore, monitoring serum ferritin levels can be used as a prognostic marker for disease severity and can aid in clinical decision-making.^6^-^9^

Maternal-fetal infection is a significant public health concern with potential long-term consequences for both the mother and the developing fetus. Early detection and treatment, along with ongoing research efforts, are crucial for improving outcomes for pregnant women and their offspring. Chorioamnionitis, a common intrauterine infection that affects both fetal membranes and the placenta, is a major cause of maternal and neonatal morbidity and mortality worldwide.^10^ In addition to in-utero fetal infections, neonatal sepsis may also result from a serious bacterial infection that can occur in the first few days of life. It is associated with significantly high mortality and long-term morbidity. Both clinical and histological chorioamnionitis are clearly associated with neonatal sepsis.^11^ However, the potential relationship between maternal serum ferritin levels and chorioamnionitis and neonatal sepsis still remains unclear.^11^,^12^

The main aim of this study was to study the implications of the changes in the levels of maternal serum ferritin in pregnancy, including its potential clinical association with maternal chorioamnionitis and neonatal sepsis. The results of our study may provide a reference direction for clinical diagnosis and treatment.

## METHODS

This is a retrospective cohort study which used clinical records of the Maternal and Child Health Hospital of Fujian Province, a maternal and child medical Center in southeastern China.

### Ethical Approval

The study was carried out from January 2014 to January 2019, and was approved by the Ethics Committee of Fujian Maternal and Child Health Hospital, Fujian Medical University (Ethical Approval No. 2022J011042).

### Inclusion criteria:

- All pregnant women who had at least one serum ferritin test performed within 12 weeks of gestation and a repeat serum ferritin test within two weeks prior to delivery.
- Signs of suspected infection prior to delivery, with at least one of the following conditions i.e increased maternal temperature (≥37.8°C), increased pulse rate (≥100 beats/min), increased fetal heart rate (≥160 beats/min), pressure at the fundus of the uterus, and odorous vaginal discharge.
- Participants with regular maternity check-ups and serum iron tests performed during early pregnancy and at admission for labor.
- Newborns admitted to the neonatal unit for examination and confirmation of diagnosis, based upon at least one blood culture.
- Neonates were followed up for two weeks.

### Exclusion criteria:

- Presence of additional iron metabolism abnormalities i.e thalassemia, autoimmune diseases.
- History of blood transfusion within a short period of time.
- Severe malnutrition, phagocytic lymphohistiocytic hyperplasia, etc.
- Multiple pregnancies.
- Cases with no placental pathologies.

All clinical diagnostic, laboratory, and delivery data were extracted from the electronic medical record system. Serum ferritin levels were measured by serum iron test using Abbott A16200 automatic biochemical analyzer. All patients had their C-reactive protein (CRP) and white blood cell (WBC) levels measured by routine blood tests at the time of the patient’s admission to hospital for labor. Alteration of Maternal Serum Ferritin in Pregnancy was calculated as late pregnancy serum ferritin minus early pregnancy serum ferritin to determine a cut-off value. Other associated details for chorioamnionitis in terms of age at delivery, pre-pregnancy body-mass index (BMI), BMI at delivery, combined gestational diabetes mellitus (GDM), and Hepatitis-B virus infection were also recorded.

For the validity of study, certain recommended definitions were taken in account. The diagnosis of chorioamnionitis was based on the histological criteria, i.e. a diffuse infiltration of neutrophils into the chorioamnion detected by microscopy.^13^ The early onset sepsis (EOS), is defined as at least one positive blood and/or cerebrospinal fluid culture at 72 hours after birth. Cultures positive for coagulase-negative staphylococci or known commensal organisms were considered to be contaminants.^14^ Early pregnancy was defined as a period from confirmation of pregnancy to 12 weeks gestation.^11^,^12^ Changes in serum ferritin during pregnancy were defined as the difference between the serum ferritin value after 28 weeks of gestation and before the delivery and the serum ferritin value in early pregnancy.^11^,^12^

### Statistical analysis

Statistical analyses were performed using R (version 4.1.2). Continuous data conforming to a normal distribution were expressed as mean ± standard deviation using the t-test, and non-normally distributed continuous data were expressed as median (interquartile spacing) using the Mann-Whitney U-test. Statistical data were expressed as n (%) and analyzed using the χ2 test, corrected chi-square test or Fisher’s exact probability method, as appropriate. Multiple logistic regression models were used to adjust for maternal comorbidities and test indicators. The association of altered serum ferritin during pregnancy with chorioamnionitis and neonatal sepsis was analyzed using the area under the receiver operating characteristic (ROC) curve (AUC) and the net reclassification index (NRI). A p-Value of < 0.05 was considered statistically significant in this study.

## RESULTS

**Fig.1:**
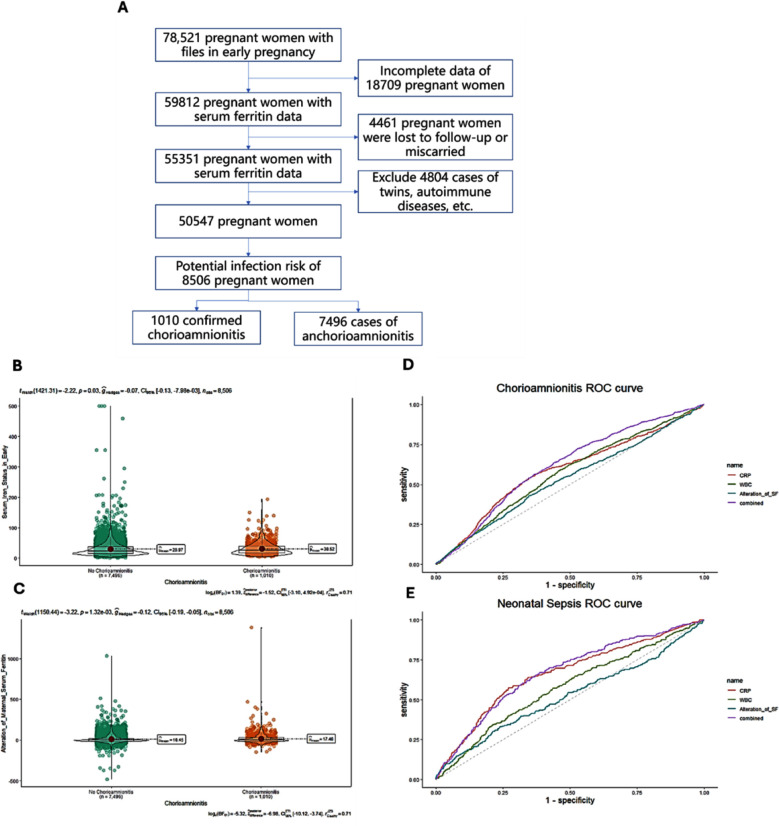
(A) Flow diagram of the participant screening. (B) Serum ferritin levels in early pregnancy; (C) Serum ferritin level in late pregnancy; (D) Receiver operating characteristic curve (ROC) curve for discrimination between the group with and without chorioamnionitis; (E) ROC curve for distinguishing neonates with and without early-onset sepsis in the control group.

Medical records of 78,521 pregnant women, admitted to the Maternal and Child Health Hospital of Fujian Province, were retrieved. Of them, records of 59,812 women with serum ferritin data were considered for inclusion in the study. As shown in detail in Fig.1A, after screening for eligibility, 8,506 pregnant women were eventually included in the study. Of them, 1010 (11.9%) had placental pathology confirming chorioamnionitis and 263 (3.1%) had premature sepsis in offspring. There were no statistical differences in the occurrence of chorioamnionitis in terms of age at delivery, pre-pregnancy body-mass index (BMI), BMI at delivery, combined gestational diabetes mellitus (GDM), and Hepatitis-B virus infection (Table-I).

**Table-I:** Clinical characteristics.

Variables	No Chorioamnionitis (n=7496)	Chorioamnionitis (n=1010)	P Value
Age at delivery (y)	28(26,32)	28(26,32)	0.338
Pre-pregnancy BMI (kg/m2)	20.31(18.8,22.21)	20.42(18.99,22.58)	0.105
BMI (kg/m2)	25.97 (24.22, 27.89)	25.85 (24.15, 28)	0.79
Gestational weeks (w)	39.29(38.29,40.03)	39.57(38,40.57)	< 0.001
Gravida, n (%)			< 0.001
G<3	5261(70)	793(79)	
3≤G<5	1946(26)	176(17)	
G≥5	289(4)	41(4)	
Parity, n (%)			<0.001
Multipartum	3687(49)	308(30)	
Primipara	3809(51)	702(70)	
PROM, n (%)			< 0.001
No	2747(37)	596(59)	
Yes	4749(63)	414(41)	
GDM, n (%)			0.475
No	6268(84)	835(83)	
Yes	1228(16)	175(17)	
Hepatitis-B, n (%)			0.856
No	6691(89)	904(90)	
Yes	805(11)	106(10)	
HDP, n (%)			0.001
No	7207 (96)	949 (94)	
Yes	289 (4)	61 (6)	
WBC (×10^9^)	9.81(8.12,12.09)	10.66(8.71,12.96)	<0.001
HGB (g/L)	118(109,127)	119(109,127)	0.817
CRP (mg/L)	3.1(1.02,14.42)	7.72(1.47,41.69)	<0.001
Delivery Method, n (%)			0.002
CS	2323(31)	362(36)	
VB	5173(69)	648(64)	
Gender of newborn, n (%)		0.126	
male	3938(53)	557(55)	
female	3558(47)	453(45)	
Stillborn Foetus, n (%)			< 0.001
No	7463(100)	968(96)	
Yes	33(0)	42(4)	
NICU, n (%)			< 0.001
No	7024(94)	784(78)	
Yes	472(6)	226(22)	
SGA, n (%)			0.01
No	7290(97)	967(96)	
Yes	206(3)	43(4)	
Neonatal sepsis, n (%)			< 0.001
No	7293(97)	950(94)	
Yes	203(3)	60(6)	

***Abbreviation:*** BMI: Body mass index, GDM: Gestational diabetes mellitus, PROM: premature rupture of membranes, WBC: White blood cell count, HGB: Hemoglobin, CRP: C-reaction protein, NICU: The Neonatal Intensive Care Unit, SGA: Small for gestational age infant.

Chorioamnionitis was more frequently diagnosed in women with slightly higher gestational age, primigravida, women with hypertensive disorders of pregnancy, and with a higher level of WBC and CRP on admission. There was no significant difference in neonatal outcome in terms of gender, but there was a correlation between chorioamnionitis and a higher rate of cesarean delivery, higher probability of stillbirth, neonatal intensive unit (NICU) admission, small for gestational age (SGA) infants and neonatal sepsis compared to women without chorioamnionitis (Table I).

Compared to normal pregnancy, women diagnosed with chorioamnionitis had higher serum iron levels (Table-II, Fig.1B) and the difference between these two groups was even more pronounced in terms of altered serum ferritin levels (Fig.1C). We calculated the Alteration of Maternal Serum Ferritin in Pregnancy (late pregnancy serum ferritin minus early pregnancy serum ferritin) to predict a cut-off value of 8.57μg/L for chorioamnionitis.

**Table-II:** Ferritin levels in early (A) and late (B) pregnancy.

A)				

Variables	No Chorioamnionitis(n=7496)	Chorioamnionitis(n=1010)	P	statistic
Serum Iron Status in Early, Median (Q1,Q3)	22.98(14.75,36.49)	25.19(16.06,39.31)	< 0.001	3502542
Serum Iron Status in Last, Median (Q1,Q3)	23.29(12.31,46.82)	28.1(15.21,59.07)	< 0.001	3362380

***B***)				

***Variables***	***No Chorioamnionitis(n=7496)***	***Chorioamnionitis(n=1010)***	***P***	***t***

Serum Iron Status in Early, mean±SD	28.97±24.22	30.52±20.41	0.0268	-2.217
Serum Iron Status in Last, mean±SD	39.42±47.26	47.98±66.82	< 0.001	-3.941

Analysis was made using nonparametric tests and t-tests.

We next performed a multiple logistic regression using the presence or absence of chorioamnionitis as the outcome variable (dependent variable). As shown in Table-IIIA, by correcting for relevant influences, all three models (models A, B and C) show that the difference in maternal serum ferritin is an independent risk factor for a final diagnosis of chorioamnionitis (p < 0.001).

**Table-III:** Adjusted ORs for Chorioamnionitis (A) and Neonatal Sepsis (B) according to Alteration of Maternal Serum Ferritin.

A)								

	Model A				Model B		Model C	

	AOR (95% CI)		P Value		AOR (95% CI)	P Value	AOR (95% CI)	P Value
Chorioamnionitis								
Alteration of Serum Ferritin	1.023 [1.012, 1.035]		<0.001		1.026 [1.015, 1.039]	<0.001	1.027 [1.015, 1.039]	<0.001

Model A: adjustment was made for alteration of maternal serum ferritin (10-unit increments), Age at delivery, pre-pregnancy BMI.								
Model B: AOR were additionally adjusted for made for Age at delivery, pre-pregnancy BMI, PROM, GDM.								
Model C: adjustment was made for Age at delivery, pre-pregnancy BMI, PROM, GDM, CRP, WBC.								

***B)***								

	***Model A***			***Model B***			***Model C***	

	***AOR (95% CI)***		***P Value***		***AOR (95% CI)***	***P Value***	***AOR (95% CI)***	***P Value***

Neonatal Sepsis								
Alteration of Serum Ferritin	0.989 [0.962, 1.014]		0.40		0.991 [0.964, 1.016]	0.508	0.994 [0.967, 1.018]	0.652

Model A: Adjustment was made for alteration of maternal serum ferritin (10 unit increments), Age at delivery, pre-pregnancy BMI. Model B: AOR were additionally adjusted for made for Age at delivery, pre-pregnancy BMI, PROM, GDM. Model C: Adjustment was made for Age at delivery, pre-pregnancy BMI, PROM, GDM, CRP, WBC.

Multiple logistic regression with the occurrence of preterm sepsis in the neonate as the outcome variable (dependent variable) demonstrated that by correcting for relevant influences, all three models (models A, B and C) show a lack of statistically significant correlation between maternal serum iron alterations during pregnancy and the occurrence of preterm sepsis in the neonate (Table-IIIB, p > 0.05).

Maternal serum iron alteration during pregnancy alone predicted an AUC of 0.532 for chorioamnionitis, compared to an AUC of 0.586 for the conventional indicator CRP and 0.568 for white blood cell count (Fig. 1D). A combination of the three factors predicted an AUC of 0.615. Maternal serum iron during pregnancy altered the NRI by 12.18% (p = 0.00014).

The AUC for maternal serum iron change as a single metric predictor of neonatal early-onset sepsis was 0.521, significantly lower than the AUC for CRP and white blood cell count (0.662 and 0.576, respectively) (Fig.1E).

## DISCUSSION

This study showed that maternal serum ferritin level is an independent risk factor for chorioamnionitis but does not correlate with the incidence of neonatal sepsis. Iron is closely associated with infection status. However, serum ferritin levels, an important indicator of iron, vary widely in the population. Our retrospective cohort study found a correlation between increased maternal serum ferritin changes during pregnancy and the occurrence of chorioamnionitis. However, we showed that maternal serum ferritin changes were not an independent risk factor for early-onset sepsis. Elevated serum ferritin may be involved in a number of pathological processes including infections, tumors and premature birth,^7^,^15^-^17^ but only few studies have previously confirmed a possible correlation between elevated maternal serum ferritin and chorioamnionitis.^18^ Chorioamnionitis is difficult to diagnose histologically prior to delivery. While common indicators, such as CRP, are used for assessing the risk of chorioamnionitis, their predictive efficiency is low.^19^ Therefore, changes in serum ferritin may serve as a good complementary indicator. Moreover, the combined prediction of these two indicators has proven to be feasible in hematopoietic stem cell transplantation.^20^

This may be related to an increase in plasma non-transferrin-bound iron (NTBI) leading to susceptibility to infection.^21^pancreas, and other organs. However, whether it could enter the small intestine and its effects still remain unclear. Herein, these issues were explored. Mice were intravenously administrated of ferric citrate (treatment Some studies also suggest a positive correlation between serum ferritin levels and WBC, and show that the combination of these common clinical indicators does improve to some extent their predictive ability in patients with chorioamnionitis.^6^ A significant clinical implication of our finding is that it may lead to earlier detection of chorioamnionitis, which could increase the treatment window and reduce the occurrence of serious complications for both mother and fetus. In addition, understanding the role of serum ferritin in the pathogenesis of chorioamnionitis may be used for new therapeutic approaches. Our study calculated a cut-off value of 8.57μg/L for chorioamnionitis. However, more high-qulaity studies are needed to determine the optimal threshold for screening and to explore the possible pathophysiological mechanisms.

Our study found little association between maternal serum ferritin and neonatal sepsis. This may be because the clinical symptoms were not stratified in this study, and chorioamnionitis was only diagnosed by histopathology. A number of studies have confirmed that peripheral blood serum ferritin may be a biomarker for the severity of sepsis in children.^22^,^23^ Previous studies have shown that there is a significant correlation between clinical symptoms and neonatal prognosis in patients with chorioamnionitis.^24^the first definition requiring maternal fever alone (Fever This poor association may also be related to the complexity of passing the infection from mother to fetus. The type, titer and antigenicity of the microorganism, the site of infection, and the maternal immune response may all determine different disease progression and pregnancy outcomes.^10^

Although early-onset neonatal infections are acquired before or during delivery, the most common source of infection is vertical transmission from mother to child.^25^ In addition, numerous studies show that maternal chorioamnionitis is a significant risk factor for early-onset neonatal infection.^11^,^26^ However, chronic and acute intrauterine inflammation signs, detected by placental pathology, may only represent exposure of the fetus to infectious conditions rather than the occurrence of actual inflammation.^25^ Furthermore, although peripheral blood currently provides serum iron to the fetus via the placenta, sepsis may occur through the caspase-11-GSDMD pathway and associated cellular scorching leading to serum ferritin release in vivo and in vitro.^27^

Therefore, elevated serum ferritin may be a result of inflammatory infection rather than the cause. At the same time, it has also been found that in inflammatory infections, serum ferritin changes are not good predictors of infectious disease prognosis,^28^ which reveals the complexity of this process. Additionally, it is important to note that over half of mothers with vaginal Group B streptococcus colonization, a common status in pregnant women, will transmit the infection during labor with subsequently increased risk of neonatal sepsis that is not related to the inflammation of placenta,^29^ and therefore, would not be associated with altered ferritin levels.

Our results revealed that the incidence of PROM was lower in the group of women with chorioamnionitis compared to women without this condition. Previous research reported a significant increase in serum ferritin in pregnant women with premature rupture of membranes (PROM).^30^,^31^ However, studies show that the incidence of histological chorioamnionitis is inversely related to gestational age at birth and directly correlates with the duration of fetal membrane rupture.^32^ Therefore, chorioamnionitis in cases of preterm labor may present solely as preterm labor with no other signs of infection.^33^ We may speculate that this inconsistency results from the fact that our study included pregnant women with suspected infection, and premature rupture of membranes (PROM) is considered a high-risk factor for underlying infection.

### Limitations of the study

This is a retrospective study. The PROM was over-represented in our study, causing bias. In addition, this study included only patients near full-term gestational age. Due to access issues, data on medication (iron supplementation) during pregnancy could not be retrieved and therefore the study was unable to provide any conclusions regarding whether iron supplementation treatment increased chorioamnionitis. Additionally, although serum ferritin levels were elevated in patients with chorioamnionitis, a precise cut-off level of ferritin for accurate prediction may be difficult to determine.

## CONCLUSION

Our study revealed that maternal serum ferritin changes during pregnancy were predictive of chorioamnionitis but did not correlate with neonatal sepsis. However, due to access issues, data on medication (iron supplementation) during pregnancy could not be retrieved and therefore the study was unable to provide any conclusions regarding whether iron supplementation treatment increased chorioamnionitis.

### Authors’ contributions:

**XL:** Conceived and designed the study.

**XX, GX, LW, ZF and HH:** Collected the data and performed the analysis.

**XL:** Was involved in the writing of the manuscript and is responsible for the integrity of the study.

All authors have read and approved the final manuscript.
